# Multivoxel Patterns in Fusiform Face Area Differentiate Faces by Sex and Race

**DOI:** 10.1371/journal.pone.0069684

**Published:** 2013-07-31

**Authors:** Juan Manuel Contreras, Mahzarin R. Banaji, Jason P. Mitchell

**Affiliations:** Department of Psychology, Harvard University, Cambridge, Massachusetts, United States of America; Tel Aviv University, Israel

## Abstract

Although prior research suggests that fusiform gyrus represents the sex and race of faces, it remains unclear whether fusiform face area (FFA)–the portion of fusiform gyrus that is functionally-defined by its preferential response to faces–contains such representations. Here, we used functional magnetic resonance imaging to evaluate whether FFA represents faces by sex and race. Participants were scanned while they categorized the sex and race of unfamiliar Black men, Black women, White men, and White women. Multivariate pattern analysis revealed that multivoxel patterns in FFA–but not other face-selective brain regions, other category-selective brain regions, or early visual cortex–differentiated faces by sex and race. Specifically, patterns of voxel-based responses were more similar between individuals of the same sex than between men and women, and between individuals of the same race than between Black and White individuals. By showing that FFA represents the sex and race of faces, this research contributes to our emerging understanding of how the human brain perceives individuals from two fundamental social categories.

## Introduction

One of the seminal breakthroughs in cognitive neuroscience was the discovery of a region of fusiform gyrus that responds preferentially to human faces, dubbed fusiform face area [FFA; 1,2]. FFA is thought to extract the physical information that distinguishes the faces of different people; that is, to represent face identity (for review, see [3]). Familiar faces elicit more neural activity in FFA than unrecognized faces [4], and lesions to FFA impair face recognition [5]. Moreover, experiments using neural adaptation–in which repeated presentation of a stimulus property decreases neural activity in brain regions that represent the property [6]–suggest that FFA is more sensitive to changes in face identity than to physical changes unrelated to face identity [7], [8]; cf. [9], [10].

But it is impossible to identify people by their faces without accurately categorizing their sex and race. The sex and race of a face determine how its identity is represented, inextricably linking face identity to these two social categories (for review, see [11]). Indeed, face morphology shows pronounced sexual dimorphism and racial differences [12], [13]. Recently, a set of studies have used multivariate pattern analysis (MVPA) to investigate whether fusiform gyrus represents the sex and race of faces. Univariate data analyses average the responses of multiple voxels. This spatial averaging reduces the information content of the data, which can exist at the level of the individual responses of multiple voxels, or *multivoxel patterns* [14]. In contrast, MVPA interrogates these patterns to reveal the representations that a brain region contains (for review, see [15]). For example, a brain region in which faces of men and women elicit distinct multivoxel patterns but faces of the same sex yield similar patterns may represent sex.

Two studies have suggested that fusiform gyrus represents the sex and race of faces. In one study, participants in a functional magnetic resonance imaging (fMRI) scanner viewed faces of famous and unfamiliar men and women [16]. Pattern classifiers decoded the sex of the faces from fusiform gyrus. In another study, participants were scanned while viewing faces of unfamiliar Black and White individuals [17]. Pattern classifiers decoded the race of the faces from fusiform gyrus. However, the sex finding has not been tested in FFA and the race finding has not been replicated reliably in FFA. Multivoxel patterns in FFA from participants who viewed the faces of Black and White individuals differentiated faces by race only for participants who showed high anti-Black bias [18]. A different study in which participants viewed photographs of Asian and White faces found that multivoxel patterns in FFA cannot distinguish faces by race [19]. Therefore, these studies suggest that fusiform gyrus may represent sex and race. However, evidence on whether FFA represents race is mixed (one negative result and one qualified positive result) and no study of which we are aware has examined whether FFA represents sex.

Additionally, the studies that decoded social categories from fusiform gyrus [16], [17], [18] have an important limitation. They did not equate physical differences between photographs of social categories that were unrelated to their facial structure, such as luminance and contrast as well as high-level differences like hair length. Consequently, the distinct patterns associated with social categories may not have reflected face differences. Consistent with this concern, the pattern classifiers in these studies decoded the social categories of faces in early visual cortex, which is not face-selective.

The present experiment continues the study of race representations in FFA and begins the study of sex representations in this face-selective brain region by scanning participants while they categorized faces of unfamiliar Black men, Black women, White men, and White women by sex and race. The goal of the present experiment is to determine if, despite the significant variability in the appearance of the people in the photographs, distinct pattern of voxels represent female and male faces as well as Black and White faces, suggesting that FFA includes representations of such social category information. We avoid the important limitation of insufficiently-controlled stimuli in two ways. First, we used photographs that are uniform in appearance and emotional expression, cropping face-irrelevant features (e.g., hairstyle) and background. Also, we controlled for low-level visual differences by equalizing luminance and contrast across social categories. Second, our stimuli orthogonalize sex and race so that if FFA differentiates faces by sex *and* race, this is unlikely to be caused by photograph differences unrelated to facial structure.

## Method

### Participants

Participants provided their written informed consent in a manner approved for this study by the Committee on the Use of Human Subjects in Research at Harvard University, which specifically approved this study. Seventeen college students and community members from Cambridge, MA, participated in this study (9 female; age range 18–34, *M* = 22.18). All participants were right-handed, had no history of neurological problems, and described themselves as White.

### Stimuli and Behavioral Procedure

In a *categorization task*, participants viewed 192 photographs of unfamiliar Black men, Black women, White men, and White women (48 photographs in each condition). Because previous research is limited by insufficient stimuli control, the present stimuli were meticulously standardized to rule out alternative interpretations of any results. Photographs were collected from a variety of different online databases and depicted young adults facing forward with mouths closed, neutral expression, and eye gaze directed at the camera. The photographs were grayscaled and cropped to squares, their background was removed, and the luminance and contrast of the faces were equalized across conditions using in-house MATLAB code (MathWorks, Natick, MA). For example, the grayscaled images of Black and White faces differed in luminance, measured in 8-bit RGB integers (*M*_Blacks_ = 106.67, *M*_Whites_ = 144.52), *t*(95) = 8.11, *p*<10^−12^, but preprocessing removed this difference (*M*_Blacks_ = 130, *M*_Whites_ = 130).

In each scanning run, participants categorized the faces either by sex (man, woman) or by race (Black, White) using the index and middle fingers of their right hand, which rested on a button box. Each run was pseudorandomly assigned a categorization dimension (sex, race). Before each run, participants were instructed as to which categorization dimension (sex or race) to use and which button would correspond to each social category. Then, participants completed 10 practice trials on a set of 10 faces not used in the categorization task. Across runs, we counterbalanced the button assignments in such a way that each social category was assigned to each finger an equal number of times and each photograph was categorized once with the index finger and once with the middle finger.

Each trial lasted 2000 ms. For the first 500 ms, a photograph was shown in the center of the screen. For the remaining 1500 ms of each trial, the photograph was replaced with a white fixation crosshair, which encouraged participants to attend to the photographs closely. Photographs were segregated into 8 runs, each of which consisted of 48 photographs (12 in each of the four social categories, e.g., Black men). To optimize estimation of the event-related fMRI response, trials were intermixed in a pseudorandom order and separated by a variable stimulus interval (0–10 s) during which participants passively viewed a white fixation crosshair in the center of the screen [20].

After the categorization task, participants completed two runs of a canonical *face localizer* used to identify cortical regions responsive to faces [1]. In each run, participants viewed photographs of human faces, human bodies, scenes, household objects, and scrambled versions of the household objects. Each photograph appeared for 1 s and was followed by a blank screen for 333 ms. Each category was blocked together to yield 10 blocks of 11 photographs each, 2 blocks per category. One photograph in each block was presented twice in a row, and participants were instructed to press a button when they detected this repetition. The blocks were separated by a stimulus interval that lasted 12 s and were presented in a pseudorandom order, such that participants could not anticipate the category of the upcoming block. During the task, participants fixated on a small, black circle that appeared in the center of the screen throughout the entire experiment (including the presentation of the photographs).

### Functional Imaging Procedure

Imaging data were acquired on a 3.0 Tesla Siemens Tim Trio scanner (Siemens, Erlangen, Germany) with a standard head coil at the Center for Brain Science at Harvard University. Functional runs used a gradient-echo, echo-planar pulse sequence (TR = 3000 ms; TE = 28 ms; flip angle = 85°; field of view = 216×216 mm; matrix = 72×72; in-plane resolution = 2.5×2.5 mm; slice thickness = 2.5 mm). Forty-five interleaved axial slices parallel to the AC-PC line were obtained to cover most of the cerebrum; portions of superior parietal lobe were not covered. The categorization task consisted of 8 runs of 43 volume acquisitions each and the face localizer consisted of 2 runs of 98 volume acquisitions each. Each of the functional runs was preceded by 8 s of gradient and radio frequency pulses that allowed the scanner to reach steady-state magnetization. After the functional runs in the experiment, a high-resolution T1-weighted structural scan (MEMPRAGE) was conducted.

### Functional Imaging Data Analysis

#### Univariate analyses

FMRI data were preprocessed and analyzed using Statistical Parametric Mapping 8 (SPM8; Wellcome Department of Cognitive Neurology, London, United Kingdom) and in-house MATLAB code (MathWorks, Natick, MA) written by Dylan Wagner (Dartmouth College, Hanover, NH). To correct for head movement, a rigid-body transformation realigned images within each run and across all runs using the first functional image as a reference. Realigned images were unwarped to reduce any additional distortions caused by head movement. Unwarped data were normalized into a stereotaxic space (2-mm isotropic voxels) based on the SPM8 EPI template that conforms to the ICBM 152 brain template space and approximates the Talairach and Tournoux atlas space. Normalized images were spatially smoothed using a Gaussian kernel (8-mm full-width-at-half-maximum) to maximize signal-to-noise ratio and reduce the impact of individual differences in functional neuroanatomy. Finally, individual runs were analyzed on a participant-by-participant basis to find outlier volumes with Artifact Detection Toolbox (ART; McGovern Institute for Brain Research, Cambridge, MA). Outliers were defined as volumes in which participant head movement exceeded 0.5 mm or 1° and volumes in which overall signal were more than three standard deviations outside the mean global signal for the entire run.

For each participant, a general linear model (GLM) was constructed to include task effects and nuisance regressors (run mean, linear trend to account for signal drift over time, six movement parameters computed during realignment, and, if any, outlier scans identified by ART and trials in which participants did not provide a response). To compute unweighted (*β*) and weighted (*t*) parameter estimates for each condition at each voxel, the GLM was convolved with a canonical hemodynamic response function (HRF). The GLM of the categorization task was also convolved with the temporal and spatial derivatives of the HRF, which explain a significant portion of BOLD variability above and beyond the canonical model in event-related designs [21]. Trials were modeled as events of durations equal to their respective reaction times to account for differences in response times (RTs) across conditions [22].

Comparisons of interest were implemented as linear contrasts. In the categorization task, linear contrasts identified significant voxels with a voxel-wise statistical criterion of *p*<.005. Regions-of-interest (ROIs) were required to exceed 75 voxels in extent, establishing an experiment-wide statistical threshold of *p*<.05, corrected for multiple comparisons, on the basis of Monte Carlo simulations [23]. In the face localizer, ROIs were identified for each participant with a voxel-wise statistical criterion of, at most, *p*<.05 (median *p* = .005). Additional statistical comparisons between conditions were conducted in MATLAB using ANOVA on the parameter estimates associated with each trial type.

#### Multivariate analyses

Preprocessing and GLM estimation were identical to those for the univariate analysis of the face categorization task, except that normalized images were spatially smoothed using a smaller Gaussian kernel (5-mm full-width-at-half-maximum).

Trials were conditionalized by sex (men, women), race (Black, White) and run type (odd, even) to yield eight conditions (e.g., *Black men-even*). Linear contrasts compared each condition to baseline. Following Misaki, Kim, Bandettini, and Kriegeskorte [24], these parameter estimates were used for the rest of the analysis to reduce the influence of noisy voxels. The parameter estimates were extracted from each of the ROIs defined by the face localizer and correlated in three ways: same-sex correlations (*Black men-odd with White men-even*, *Black men-even with White men-odd*, *Black women-odd with White women-even*, *Black women-even with White women-odd*), same-race correlations (*Black men-odd with Black women-even*, *Black men-even with Black women-odd*, *White men-odd with White women-even*, *White men-even with White women-odd*), and different-category correlations (*Black men-odd with White women-even*, *White men-odd with Black women-even*, *Black women-odd with White men-even*, *White women-odd with Black men-even*).

Correlations were Fisher-transformed to *z*-values and averaged to yield one same-sex correlation, one same-race correlation, and one different-category correlation. Then, the different-category correlation was subtracted from each of the other average correlations to yield two correlation differences. Finally, one-tailed, one-sample *t*-tests determined if these correlation differences were reliably greater than zero across participants.

## Results

### Behavioral Data

Table 1 displays means and standard deviations of responses and RTs. Participants categorized faces more accurately and more quickly by sex (*M*_accuracy_ *= *0.98, *M*_RT_ *= *670 ms) than race (*M*_accuracy_ *= *0.95, *M*_RT_ *= *712 ms), *ts*(16) >5.65, *ps* <10^−5^, *Cohen’s d*s >1.41. Participants categorized men (*M*_accuracy_ *= *0.97, *M*_RT_ *= *684 ms) more accurately and more quickly than women (*M*_accuracy_ *= *0.96, *M*_RT_ *= *699 ms), *ts*(16) >2.25, *ps* <.04, *d*s >0.56. Although participants were no more accurate to categorize Black (*M*_accuracy_ *= *0.96) than White faces (*M*_accuracy_ *= *0.96), *p* = .15, they were faster to categorize Black (*M*_RT_ *= *683 ms) than White faces (*M*_RT_ *= *699 ms), *t*(16) = 3.05, *p*<.01, *d* = 0.76. The sex and race of photographs did not interact in participants’ accuracy and RT, whether collapsing across sex and race runs, within sex runs, or within race runs, all *p*s >.22. Moreover, the 3-way interaction of photograph sex, photograph race, and run (sex, race) was not statistically reliable for accuracy and RT, all *p*s >.28.

**Table 1:** Participants’ responses and response latencies from the categorization task.

	Accuracies		Response Latencies	
	Sex	Race	Sex	Race
White men	0.95^acd^ (0.04)	0.98^bd^ (0.02)	706^acd^ (65)	679^bd^ (64)
White women	0.94^cdd^ (0.05)	0.97^ad^ (0.03)	722^cdd^ (86)	692^ab^ (78)
Black men	0.96^acd^ (0.04)	0.98^bd^ (0.03)	700^acd^ (60)	650^ed^ (65)
Black women	0.94^c d^ (0.05)	0.98^bd^ (0.02)	722^ddd^ (67)	661^fd^ (55)

Note: Means and, in parentheses, standard deviations. Accuracies are displayed in proportions of correct categorizations. Response times are displayed in milliseconds. For each dependent variable, means sharing a superscript do not differ significantly at *p*<.05, as computed in paired-samples *t*-tests.

### Functional Imaging Data

#### Univariate analyses

The face localizer was used to identify FFA and control brain regions independently (Table 2). Replicating previous research [1], [2], the contrast of *faces*>[*bodies+scenes+objects+scrambled objects*] identified a bilateral region of fusiform gyrus that corresponds to FFA. As face-selective control regions, this contrast also identified a bilateral region of inferior occipital gyrus that corresponds to occipital face area (OFA) [25], and a bilateral region of superior temporal sulcus (STS) [26]. As control regions that are category-selective but not face-selective, the contrast of *scenes*>*objects* identified a bilateral region of parahippocampal gyrus that corresponds to parahippocampal place area (PPA) [27]. Additionally, the contrast of *objects*>*scrambled objects* identified a bilateral region of lateral occipital cortex that corresponds to lateral occipital complex (LOC) [28].

**Table 2:** Brain regions identified in whole-brain, random-effects contrasts in the categorization task, *p*<.05, corrected for multiple comparisons.

Faces>[Bodies+Scenes+Objects+Scrambled Objects]				
*Region*	*x*	*y*	*z*	Participants
Fusiform gyrus (FFA)	38.8	−44.3	−18.5	16
	−37.1	−47.6	−17.3	16
Inferior occipitalgyrus (OFA)	33.3	−76.7	−8.9	14
	−33.1	−77.0	−6.55	11
Superior temporalsulcus (STS)	49.8	−43.4	13.9	16
	−49.8	−52.8	21.3	9

Scenes>Objects				
*Region*	*x*	*y*	*z*	Participants
Parahippocampalgyrus (PPA)	23.4	−39.5	−7.4	16
	−24.1	−42.9	−4.8	16

Objects>Scrambled Objects				
*Region*	*X*	*y*	*z*	Participants
Lateral occipitalcortex (LOC)	40.5	−66.3	−5.0	8
	−42.0	−63.7	−6.7	10

Note: From left to right, columns list the names of regions obtained from whole-brain, random-effects contrasts, the mean stereotaxic Montreal Neurological Institute coordinates of their peak voxels across participants, and the number of participants (*N* = 17) in whom these brain regions were identified at *p*<.05, corrected for multiple comparisons. FFA = fusiform face area, OFA = occipital face area, STS = superior temporal sulcus, PPA = parahippocampal place area, LOC = lateral occipital complex.

For completeness, univariate analyses of the categorization task examined potential differences between photographs as a function of their sex and race. For these analyses, trials were conditionalized by sex (men, women) and race (Black, White; Table 3).

**Table 3:** Brain regions identified in whole-brain, random-effects contrasts in the face localizer task, *p*<.05, corrected for multiple comparisons, sorted in descending order by the *t*-statistic of their peak voxel (*t*).

Men>Women					
No brain regions identified.					
Women>Men					
*Region*	*x*	*y*	*z*	*k*	*t*
Cerebellum	0	−61	−16	204	5.18
Inferior frontal gyrus	−28	15	−20	231	4.71
Superior frontal gyrus	20	61	−6	89	4.50
Cingulate gyrus	4	−29	34	75	3.99

White>Black					
*Region*	*x*	*y*	*z*	*k*	*t*
Middle frontal gyrus	−16	33	−8	437	7.73
	14	35	−12	162	6.06
Cerebellum	−12	−57	−32	82	5.08
Cingulate gyrus	−20	−31	44	112	4.83
Precuneus	−16	−45	22	105	4.12

Black>White					
*Region*	*x*	*y*	*z*	*k*	*t*
White matter	−18	−81	2	126	5.36
Supramarginal gyrus	48	−53	34	142	4.60

Note: From left to right, columns list the names of regions obtained from whole-brain, random-effects contrasts, the stereotaxic Montreal Neurological Institute coordinates of their peak voxels, their size in number of voxels (*k*), and the *t*-statistic of their peak voxel (*t*).

#### Multivariate analyses

**Figure 1:**
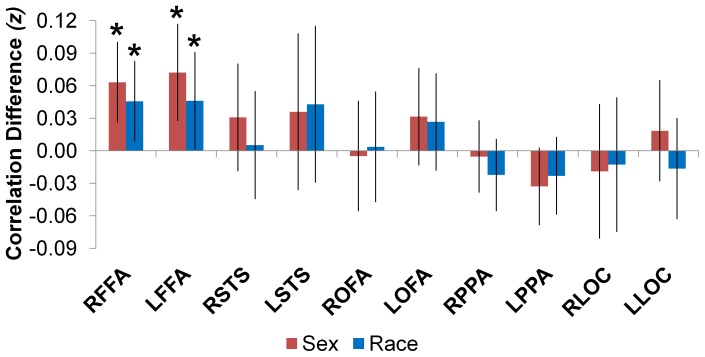
Bar graphs display mean correlation differences expressed in *z*-scores (*same-sex>different-category* in red, *same-race>different-category* in blue). An asterisk denotes a correlation difference that is reliably greater than zero across participants, *p*<.05. Error bars represent 95% confidence intervals in within-subject comparisons [39]. R and L as the first letters of a region-of-interest’s (ROI) acronym denote the brain hemisphere in which the ROI is localized. FFA = fusiform face area, OFA = occipital face area, STS = superior temporal sulcus, PPA = parahippocampal place area, LOC = lateral occipital complex.

First, we examined whether FFA maintains distinct representations of female and male faces; that is, whether multivoxel patterns in FFA show higher correlations between photographs of individuals of the same sex than between photographs of men and women (Figure 1). Consistent with the hypothesis that FFA distinguishes faces by sex, pattern correlations in FFA were higher between photographs of the same sex than between photographs of men and women (right FFA, *t*(15) = 3.03, *p*<.005, Cohen’s *d* = 0.78; left FFA, *t*(15) = 2.73, *p*<.008, Cohen’s *d* = 0.70). The correlation differences of right and left FFA were equivalent, *t*(14) = 0.69, *p* = 0.50, suggesting that both regions distinguished faces by sex to a similar degree.

Second, we examined whether FFA maintains distinct representations of Black and White faces; that is, whether multivoxel patterns in FFA show higher correlations between photographs of individuals of the same race than between photographs of Black and White individuals (Figure 1). Consistent with the hypothesis that FFA distinguishes faces by race, pattern correlations in FFA were higher between photographs of the same race than between photographs of Black and White faces (right FFA, *t*(15) = 1.72, *p* = .05, Cohen’s *d* = 0.44; left FFA, *t*(15) = 2.21, *p*<.02, Cohen’s *d* = 0.57). The correlation differences of right and left FFA were equivalent, *t*(14) = 1.01, *p* = 0.33, suggesting that both regions distinguished faces by race to a similar degree.

The correlation differences that suggest distinct representations of female and male faces and Black and White faces in FFA are statistically reliable with a small sample, although they are not corrected for multiple comparisons (Figure 1). However, the corresponding effect sizes are not small. The correlation differences that correspond to sex representations have effect sizes that approach a large effect size (Cohen’s *d* = 0.8) [29], whereas the correlation differences that correspond to race representations have effect sizes that hover around a medium effect (Cohen’s *d* = 0.5) [29].

We speculated that FFA might be the only face-selective brain region to represent the sex and race of faces because it is the face-selective region that is most sensitive to face identity [3]. To test this hypothesis, we repeated the MVPA with patterns extracted from other brain regions defined by the face localizer, which included ones previously implicated in face processing like OFA and STS [3] (Figure 1). Neither right nor left OFA or STS distinguished faces by social category reliably, *p*s >.13. This suggests that FFA is alone among face-selective brain regions in decoding the sex and race of faces. Because face information may exist in category-selective cortex outside of FFA [30], [31], we repeated the pattern similarity analyses with patterns extracted from place-selective PPA and object-selective LOC (Figure 1). Neither right nor left PPA or LOC distinguished faces by social category reliably, *p*s >.26. This suggests that other category-selective brain regions lack sex and race information about faces.

However, FFA may differentiate photographs not by facial properties that vary between social categories, but by lower-level physical differences between the photographs. Many of these low-level physical differences were removed by careful photograph selection and intensive preprocessing (see *Method: Stimuli and behavioral procedure*), but we wanted to test this alternative hypothesis empirically. Therefore, we analyzed multivoxel patterns from early visual cortex, which processes lower-level visual features. To do so, we used the stereotaxic coordinates of the center of mass of the right ([*x y z*] = 25, −82, −15) and left ([*x y z*] =  −29, −80, −18) foveal confluence of brain areas V1, V2, and V3, which represents the central portion of the visual field, as functionally-defined by Dougherty *et al*. [32] using retinotopic mapping [33]. We extracted patterns from 8-mm spheres centered on these stereotaxic coordinates and repeated the pattern similarity analyses with these patterns. Neither the right nor the left foveal confluence distinguished faces by social category reliably, *p*s >.66. This suggests that low-level visual differences between the photographs do not cause multivoxel patterns in FFA to differentiate faces by sex and race.

As one more way to determine whether low-level visual differences between the stimuli resulted in distinct multivoxel patterns for faces of different social categories, information-based functional brain mapping with multivariate spherical searchlights [14] was conducted to determine if any portion of occipital lobe differentiated faces by sex or race. For each voxel in the brain, we extracted the parameter estimates of each of the eight contrasts (e.g., *Black men-even*) within a spherical neighborhood (8-mm radius; neighborhood size in resampled voxels, *M* = 254, *SD* = 11) similar in shape to those used by Kriegeskorte and colleagues [14]. For each neighborhood, a same-sex correlation difference and a same-race correlation difference were computed as before (see *Method: Functional imaging data analysis*) and assigned to the center voxel. This analysis yielded two correlation difference maps expressed in *z*-scores for each participant, indexing the degree to which each voxel exists in a neighborhood in which multivoxel patterns differentiate female from male faces (first map) and Black from White faces (second map). Finally, a univariate, random-effects analysis identified brain regions in each map that showed correlation differences reliably larger than zero across participants. For each voxel in each map, we performed a right-tailed one*-*sample *t*-test against zero with the corresponding *z*-values from all participants. Correcting for multiple comparisons (see *Method: Functional imaging data analysis*), no brain regions in occipital lobe showed distinct multivoxel patterns for female and male faces or Black and White faces (Table 4).

**Table 4:** Brain regions identified in whole-brain, random-effects contrasts from the multivariate searchlight analyses, *p*<.05, corrected for multiple comparisons.

Same-Sex>Different-Category					
*Region*	*x*	*y*	*z*	*k*	*t*
Cerebellum	18	−29	−26	77	5.20

Same-Race>Different-Category					
No brain regions identified.					

Note: From left to right, columns list the names of regions obtained from whole-brain, random-effects contrasts, the stereotaxic Montreal Neurological Institute coordinates of their peak voxels, their size in number of voxels (*k*), and their mean weighted parameter estimate (*t*).

Finally, we investigated whether participants’ task (categorization by sex or race) influenced multivoxel patterns in FFA. To do so, we tested for effects of categorization dimension in two different ways. First, trials were conditionalized by sex (men, women), race (Black, White), categorization dimension (sex, race), and run type (odd, even) to yield 16 conditions (e.g., *Black men categorized by sex-even*). The same correlation differences as before (*same-sex>different-category*, *same-race>different-category*) were calculated separately for each categorization dimension (e.g., *same-sex categorized by sex>different-category categorized by sex*). None of these correlation differences were reliably larger than zero in right and left FFA, *p*s >.16. The discrepancy between these results and the positive results of the analysis in which trials were not conditionalized by categorization dimension are most likely caused by differences in statistical power. The analysis that involves conditionalizing by categorization dimension has half as many trials per condition as the original analysis, endowing the former with an inferior ability to detect small differences between multivoxel patterns across conditions.

Second, trials were conditionalized by categorization dimension (sex, race) and run type (odd, even) to yield 4 conditions (*race-odd, race-even, sex-odd, sex-even*). We computed *same-categorization* correlations (*race-odd with race-even*, *sex-odd with sex-even*) and *different-categorization* correlations (*race-odd with sex-even*, *sex-odd with race-even*). The average different-categorization correlation was subtracted from the average same-categorization correlation to yield a correlation difference. However, this correlation difference was not reliably larger than zero in right and left FFA, *p*s >.24.

## Discussion

Previous studies suggested that fusiform gyrus represents the sex and race of faces [16], [17], although whether FFA in particular represents this information was unclear [18], [19]. In the present experiment, we observed that multivoxel patterns in bilateral FFA distinguished faces by sex and race. Participants variably categorized photographs of unfamiliar Black men, Black women, White men, and White women by sex and race. Despite the significant variability in the appearance of the people in the photographs, a distinct pattern of voxels distinguished between female and male faces and between Black and White faces, suggesting that bilateral FFA includes representations of such social category information. The differences in multivoxel patterns that suggest distinct representations of male and female faces and Black and White faces in FFA were small but statistically reliable. Moreover, their effect sizes are in a range that makes them medium to large effects [29].

These social category representations may be components of face identity representations, which are thought to exist in FFA [3]. Because face identity is inextricably linked to social categories like age, sex, and race [11], it seems reasonable that FFA might represent face identity as well as the social categories of faces. FFA could be the neuroanatomical locus in which social categories that are relevant to face identity (i.e., age, race, and sex) are integrated to form holistic representations of individual faces. This hypothesis is consistent with behavioral research that suggests that the human brain codes face identity with reference to social categories [34].

Analyses of multivoxel patterns from other brain regions suggest that representations of the sex and race of faces may be unique to FFA. Patterns extracted from other face-selective brain regions (OFA and STS), other category-selective brain regions (PPA and LOC), and early visual cortex (foveal confluence of V1, V2, and V3) did not differentiate faces by sex or race. The null results from patterns in early visual cortex suggest that the careful selection and intensive preprocessing of the stimuli removed low-level physical differences unrelated to the sex and race of the stimuli that might have existed in the original photographs. These null results are especially important in this experiment because previous studies that decoded the sex or race of faces from fusiform gyrus also decoded sex and race from early visual cortex [16], [17], [18].

FFA is thought to process perceptual rather than semantic aspects of person perception [3]; cf. [35]. For this reason, the sex and race information that FFA represents is unlikely to be semantic; that is, FFA may “tell” faces apart by sex and race without “knowing” what these differences mean. Nonetheless, FFA may play a critical role in social categorization. One of the most fruitful future directions for research on sex and race representations in FFA may be to investigate how this information guides semantic retrieval about social categories in more anterior regions of temporal lobe, which have been consistently implicated in semantics about people generally (for review, see [36]) and in stereotypes specifically [37]. Evidence exists to suggest that stereotyping can modulate neural activity in FFA [38], but how representations in FFA might inform higher-order social processes like stereotyping is unknown.

In sum, the present experiment suggests that FFA distinguishes faces by social categories like sex and race. In this way, the current research contributes to our emerging understanding of how the human brain perceives individuals from different social categories.
